# Clinical analysis of patients with acute pancreatitis complicated with hemorrhagic fever with renal syndrome and acute biliary pancreatitis

**DOI:** 10.1097/MD.0000000000018916

**Published:** 2020-01-31

**Authors:** Wen-Jie Wang, Jing Zhao, Jin-Sun Yang, Man-Man Liang, Ming-Yue Ni, Jiang-Hua Yang

**Affiliations:** a Department of Infectious Diseases; b Department of Gastrointestinal Surgery, Yijishan Hospital, Wannan Medical College, Wuhu, Anhui, China.

**Keywords:** acute biliary pancreatitis, acute pancreatitis, case analysis, clinical characteristics, hemorrhagic fever with renal syndrome

## Abstract

Acute pancreatitis (AP) is a rare complication of hemorrhagic fever with renal syndrome (HFRS), and is difficult to diagnose. In this study, we retrospectively analyzed the clinical characteristics of 7 cases of HFRS complicated with AP and 105 cases of acute biliary pancreatitis (ABP).

Medical records of 83 hospitalized patients with HFRS and 105 hospitalized patients with ABP in the affiliated Yijishan Hospital of Wannan Medical College were reviewed. The comparative analysis of patients between the 2 groups was conducted in terms of sex, age, duration of hospital stay, fever, hemorrhage, proteinuria, oliguria, laboratory results, radiologic examinations, and prognosis.

A total of 83 patients were diagnosed with HFRS during study period. Only 8.43% (7/83) of the total HFRS patients were diagnosed with AP. The differences in the gender, age, and duration of hospital stay between the 2 investigated groups of patients were not statistically significant. The major symptoms for all 7 patients with HFRS complicated with AP and 105 patients with ABP were fever and upper abdominal pain. During the disease course of HFRS complicated with AP, 6 patients experienced hemorrhaging, and 7 patients underwent an oliguric stage, but none of the ABP patients experienced hemorrhaging and oliguria. Among the laboratory results of all patients, the differences in alanine aminotransferase and glycemia were not statistically significant. The other laboratory results (leucocyte count, platelet count, amylase, lipase, total bilirubin, direct bilirubin, creatinine, blood urea nitrogen, prothrombin time, activated partial thromboplastin time, and serum calcium level) were significantly different during hospitalization. All 7 patients with HFRS complicated with AP received conservative medical treatment and hemodialysis. In the patients with ABP, 21 patients were discharged from the hospital after conservative treatment, 53 patients were treated by endoscopic invasive treatment after stabilization, and 31 patients were treated by surgery after stabilization.

AP is not a frequent complication in patients with HFRS. There are differences in clinical manifestations and laboratory findings between the HFRS complicated with AP group and the ABP group; these differences may help in the differential diagnosis and treatment of these 2 types of pancreatitis.

## 1 Introduction

Hemorrhagic fever with renal syndrome (HFRS), which occurs widely in Europe and Asia, is an acute natural immunity to infectious diseases caused by rats, ticks, and lice with Hantavirus transmission.^[1]^ The growing list of affected countries (developing and developed countries) has led to public health concerns, and patients typically present with acute kidney injury and thrombocytopenia.^[2]^ HFRS complicated with acute pancreatitis (AP), with abdominal pain as the main clinical manifestation, is not significant, and the misdiagnosis rate is very high.

The clinical manifestations of AP are characterized by gastrointestinal symptoms, including nausea, vomiting, and abdominal pain. The diagnosis of AP is based on clinical features, biochemical tests, and image studies.^[3–6]^ The most frequently implicated etiologies of AP are gallstones and alcohol abuse.^[3,5–7]^ At present, acute biliary pancreatitis (ABP), which is caused by stone obstruction, is due to an infection of the biliary tract and is the most common type of AP.

In this study, we retrospectively analyzed the clinical data, laboratory results, and prognosis of 7 cases of HFRS complicated with AP and 105 cases of ABP in our hospital and provide a review of the literature to improve the clinical recognition and management of their diagnosis and treatment.

## 2 Methods

### 2.1 Patients

This study included 83 patients with HFRS and 105 patients with ABP who were admitted to the affiliated Yijishan Hospital of Wannan Medical College. Hospital records, including the laboratory examinations, imaging data, treatment and prognosis, from January 1, 2012 to January 1, 2018 were reviewed. The clinical diagnosis of HFRS infection was confirmed by colloidal gold immunoassay tests for the detection of serum immunoglobulin IgM or IgG antibodies to Hantavirus. The tests were performed by the Center for Disease Control and Prevention of Wuhu city in the Anhui province.

The studies received the approval of the ethics committee of Yijishan Hospital, Wannan Medical College.

### 2.2 AP diagnosis

The diagnosis of AP requires 2 of the following 3 features^[8]^:

1. abdominal pain suggestive of pancreatitis (epigastric pain often radiating to the back), with the start of such pain considered to be the onset of AP;
2. serum lipase activity (or amylase activity) at least 3 times greater than the upper limit of the normal activity^[9,10]^; and
3. characteristic findings of AP on computed tomography and/or magnetic resonance images or by transabdominal ultrasonography (US).^[3,11,12]^

The selection of patients with ABP was made in accordance with the diagnostic criteria of AP; imaging examination found gallbladder stones and/or bile duct stones and laboratory examinations showed increased levels of serum alanine aminotransferase (ALT) and bilirubin.

The including criteria of HFRS complicated with AP were as follows: coincides with the diagnosis of HFRS and AP. Excluding criteria of HFRS complicated with AP were as follows: other types of injury to pancreas such as gallstones, alcohol abuse, hyperlipidemia, medication, genetic diseases, postoperative states, etc; incomplete clinical data; and cannot be followed up or lost follow-up. Including criteria of ABP were as follows: the patients were grouped in accordance with the diagnostic criteria of AP, imaging examination found gallbladder stones and/or bile duct stones, and laboratory exams showed increased levels of ALT and bilirubin. Excluding criteria were as follows: other types of injury to pancreas such as alcohol abuse, hyperlipidemia, medication, genetic diseases, infectious agents, postoperative states, endoscopic procedure involving pancreatic and bile ducts, etc; the incomplete clinical data; and cannot be followed up or lost follow-up.

### 2.3 Treatment

The main treatment during hospitalization of HFRS patients was supportive therapy, which included antiviral, management of the patient's fluid and electrolyte levels, fresh frozen plasma, platelet, albumin, and antibiotics. The HFRS patients with oliguria were given diuresis; hemodialysis was required in patients who had oliguric acute renal failure. Patients with complications with AP were given abrosia, continuous gastrointestinal decompression, omeprazole, and somatostatin. The treatment of ABP was conservative initially, including bowel rest and intravenous fluid replacement, such as abrosia, continuous gastrointestinal decompression, omeprazole, and somatostatin. The ABP patients underwent endoscopic retrograde cholangiopancreatography, endoscopic Oddi sphincterotomy, and endoscopic nasobiliary drainage after the inflammation was controlled. Some ABP patients were given surgical operation interventions, such as laparoscopic cholecystectomy or open cholecystectomy, common bile duct exploration, and T-tube drainage.

### 2.4 Prognosis judgment

The definition of complete remission is as follows: the symptoms of ABP and HFRS disease activity completely disappeared; the definition of partial remission is as follows: symptoms of ABP and HFRS disease activity can be part of the control, but not completely better. The definition of no remission is as follows: symptoms of ABP and HFRS disease activity had no obvious improvement, or the patient died.

### 2.5 Data analysis

Descriptive statistics were reported as frequencies and percentages (%) for categorical variables, and mean ± standard deviation or median (range) for continuous variables. Categorical variables were evaluated using the χ^2^ test. Normally distributed data were analyzed using Student *t* test. For non-normally distributed data, differences between groups were analyzed using Mann–Whitney *U* test. A two-tailed *P* value < .05 was considered to indicate significant difference. Data processing was carried out using SPSS 17 for Windows (version 17.0, IBM Corp., Chicago, IL).

## 3 Results

Of the 83 eligible patients who were diagnosed with HFRS from January 2012 to January 2018, only 7 (8.43%) patients developed AP. Of the 7 AP patients, 5 patients were male (71.4%) and 2 were female (28.6%). Patients ranged in age from 35 to 71 years old (average age: 48.57 ± 20.94 years). A total of 105 patients were diagnosed with ABP at our hospital during the study period. These patients comprised 57 males and 48 females between 24 and 83 years old (average age: 51.33 ± 10.79 years). Both gender and age had no statistical significance (*P* = .38 and *P* = .54, respectively). The durations of the hospital stay of HFRS complicated with AP and ABP were 16.62 ± 8.53 days and 17.77 ± 5.16 days, respectively, but they did not reach statistical significance (*P* = .59).

Forty-one (49.4%) patients with HFRS complained of abdominal pain, and the 7 patients with HFRS complicated with AP complained of constant upper abdominal pain. A total of 105 patients with ABP complained of upper abdominal pain, and 47 (44.8%) patients complained of radiating pain to the shoulder and back. After treatment, the abdominal pain gradually relieved in both groups, but the remission time (7.66 ± 1.49 days and 4.76 ± 1.23 days, respectively) was significantly different. The first symptom for all 7 cases of HFRS complicated with AP was reported to be fever, and 93 (88.6%) patients with ABP also experienced fever. Six (85.7%) patients with HFRS complicated with AP experienced clinically observed hemorrhaging during the disease course. In these patients, the hemorrhaging in 3 patients manifested as skin mucous membrane bleeding, 1 patient had gingival bleeding, 1 patient had hematuria, and 1 patient had fecal occult blood (Table 1). None of the patients with ABP had clinically observed hemorrhaging. Seven patients with HFRS complicated with AP underwent an oliguric stage with a 24-h urine volume of 200 to 400 mL, but none of the patients with ABP experienced oliguria. All patients had a semiquantitative proteinuria test, including the 7 patients with HFRS complicated with AP; the results were as follows: 4 patients +++, 2 patient ++, and 1 patient +. In the 105 patients with ABP, the semiquantitative proteinuria test results were as follows: 7 patients +, 23 patients ±, and 75 patients –. The difference in proteinuria between the 2 groups reached statistical significance (*P* < .001).

**Table 1:**
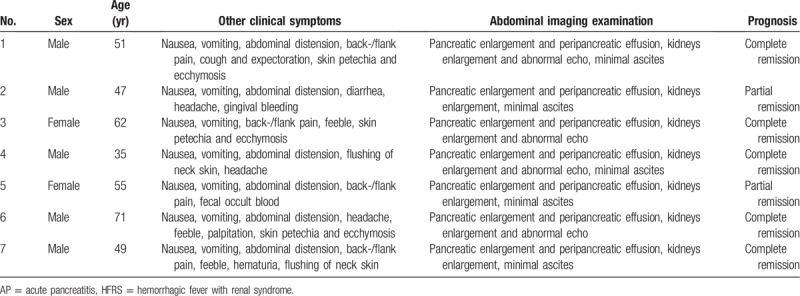
General information of 7 cases of HFRS complicated with AP.

Among all patients, the differences in ALT and glycemia levels were not statistically significant, but the other laboratory results were significantly different during their hospitalization (Table 2). Abdominal imaging studies, by computed tomography and/or magnetic resonance images, or by transabdominal US, were performed in all patients with HFRS and ABP. Seven cases of HFRS complicated with AP showed pancreatic edema or peripancreatic effusion, enlarged kidneys and abnormal echo, and minimal ascites. Abdominal imaging examinations in 105 patients with ABP revealed cholecystolithiasis (29 patients), choledocholithiasis (41 patients), or both (35 patients) and pancreatitis.

**Table 2:**
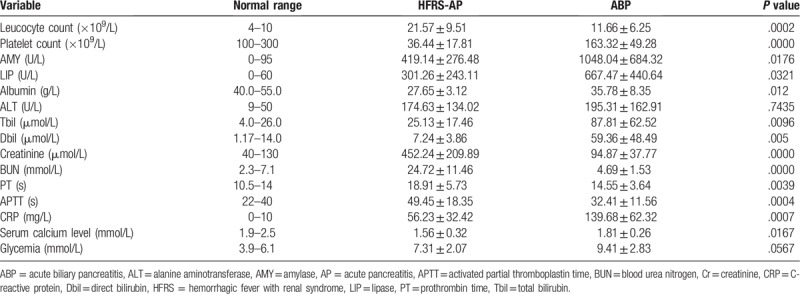
Laboratory results from all patients with HFRS complicated with AP and with ABP.

All 7 patients with HFRS complicated with AP received conservative medical treatment and hemodialysis. Upon discharge, 5 (71.4%) patients were in complete remission, 2 (28.6%) patients were in partial remission, and none of the patients died during the disease course, and no complications occurred. Meanwhile, 21 (20%) patients with ABP were discharged from the hospital after conservative treatment, 53 (50.5%) patients were treated by endoscopic invasive treatment after stabilization, and 31 (29.5%) patients were treated by surgery after stabilization. However, in the end, 4 (3.8%) patients died in the hospital during the course of the disease (1 patient died of massive acute myocardial infarction, 1 patient died of severe pulmonary infection, and 2 patients died of multiple organ failure). No statistically significant differences in prognosis were observed between the 2 groups (*P* = .78). Complications occurred in 11 (10.5%) patients in the ABP group. Among the 6 patients who had complications with pancreatic pseudocysts, 2 patients had complications with pancreatic abscess, 1 patient had complications with pancreatic necrosis, and 2 patients had complications with multiple organ failure.

## 4 Discussion

HFRS is a global public health problem, and China's epidemic situation is grim, accounting for approximately 90% of the world's cases.^[13]^ In general, after infection with Hantavirus, there is a 2- to 3-week incubation period followed by a typical 5-period clinical course, namely, febrile stage, hypotensive stage, oliguric stage, diuretic stage, and convalescent stage.^[14]^ The key feature of all stages is renal involvement; however, several extrarenal manifestations have also been reported. Park et al^[1]^ reported that one-third of patients with HFRS showed the involvement of major organs, excluding the kidney. Pancreatobiliary manifestations were the most common extrarenal manifestation in that study. Some studies from Asia, Albania, and the United States have reported that abdominal pain during the acute phase of HFRS may be caused by AP.^[2,4,15–17]^ In these studies, the AP rate during acute Hantavirus infection ranged between 2.8% and 54%. In our study, HFRS complicated with an AP incidence rate was 8.43% (7/83), which is consistent with the report of Fan et al^[16]^ The male incidence rate was higher than that of females, which should be given more attention. The basic pathogenesis of HFRS is the leakage of small blood vessels and capillaries,^[2]^ which can affect multiple organs. During the infection, the patient's capillaries become engorged, and focal hemorrhages develop; ultimately, the systemic expansion of capillary leakage leads to retroperitoneal edema, which may affect the pancreas.^[1]^ Potential mediators, which increase vascular permeability during the acute stage of HFRS, are tumor necrosis factor-alpha, interleukin-1 and interleukin-2, and nitric oxide.^[18]^ Activation of inflammation mediators during the hypotensive stage of HFRS seems to be caused by an inflammatory cascade mediated by cytokines, immunocytes, and the complement system. Inflammatory cytokines cause macrophages to migrate into tissues distant from the pancreas, including the lungs and kidneys.^[18]^ This may be the possible pathogenic mechanisms of AP in HFRS. Abdominal pain is a common symptom of Hantavirus infection, with an estimated 46% of patients presenting with this as their initial complaint or experiencing it at some time during their hospitalization.^[4]^ In our study, 41 (49.4%) patients with HFRS complained of abdominal pain, and 7 patients of HFRS complicated with AP complained of constant upper abdominal pain. A total of 105 patients with ABP complained of upper abdominal pain, and 47 (44.8%) ABP patients complained of radiating pain to their shoulder and back. Thus, pancreatitis is easily misdiagnosed as acute cholecystitis, acute gastrointestinal perforation, or acute intestinal obstruction. Only for these secondary disease-appropriate treatment measures, ignoring HFRS is the primary cause of the disease. The main reasons for misdiagnosis include physicians are not familiar with HFRS, depend too much on the auxiliary examinations, and lack the whole thinking and comprehensive analysis of data.

The typical pathological features of HFRS are systemic capillary and small vessel injury, vascular paralysis, dilatation, increased fragility and permeability, and capillary rupture, and results in massive plasma extravasation, extensive tissue edema and hemorrhage, microthrombosis, and inflammatory cell infiltration. All of these lead to typical clinical symptoms of HFRS. ABP results from the migration of a gallstone to the common bile duct with impaction or temporary obstruction of the major duodenal papilla.^[9]^ The diagnosis of ABP should be suspected if the patient has a prior history of biliary colic.^[19,20]^ In our study, 7 patients with HFRS complicated with AP experienced fever during the disease course, and 93 patients with ABP experienced fever. There was no significant difference between the 2 groups regarding experienced fever. Compared with the ABP group, the symptoms of hemorrhaging, oliguria, and proteinuria, which are distinguished features from ABP and general AP, were significantly higher in the HFRS complicated with AP group, and the difference was statistically significant. All patients in both groups experienced abdominal pain, but compared with the ABP group, the HFRS complicated with AP group required a longer time to relieve abdominal pain, and the difference was statistically significant. It is speculated that the cause of abdominal pain in HFRS patients may be due to the invasion of the Hantavirus and the immune response. Additionally, the abdominal pain in ABP patients was relieved after the obstructive factors were removed.

Compared to patients with ABP, leukocyte elevation and thrombocytopenia were more notable in patients with HFRS complicated with AP, and renal function indexes, such as creatinine and blood urea nitrogen, were significantly higher in patients with HFRS complicated with AP than in ABP patients. The prothrombin time and activated partial thromboplastin time were prolonged significantly in HFRS complicated with AP and are associated with thrombocytopenia and immune response caused by the Hantavirus infection. The above indicators can be used as the basis for the identification of HFRS complicated with AP and ABP. Compared to patients with HFRS complicated with AP, the levels of pancreatic enzymes (amylase and lipase) were greater in patients with ABP, and the difference was statistically significant. The 2 groups of patients can lead to liver function damage, but there was no significant difference in the elevation of ALT; however, the elevation of total bilirubin and direct bilirubin was statistically significant. A possible explanation for this is that ABP induced biliary obstruction and cholestasis. Albumin levels in both groups decreased, especially in the group of patients with HFRS complicated with AP, possibly due to severe renal impairment and protein excretion from urine in the group of patients with HFRS complicated with AP. Recently, as the roles of inflammatory response and oxidative stress in the pathogenesis of AP have emerged, inflammatory markers have been proposed as better predictors of disease severity. The most promising of these are C-reactive protein (CRP), interleukin-6, and, in the urine, albumin, immunoglobulin, and trypsinogen activation peptide.^[21–25]^ Kaya et al^[26]^ showed that although HFRS was caused by a viral infection, CRP levels in these patients were elevated. This study showed that both patients with HFRS complicated with AP and with ABP could cause elevated levels of CRP, but compared to the group with HFRS complicated with AP, the CRP elevation in the ABP group was higher; the difference was statistically significant, and, presumably, the cause may be the inflammation reaction in patients with ABP. This study showed that both groups of patients can cause hypocalcemia, but compared to patients with ABP, patients with HFRS complicated with AP had lower calcium levels in the blood, which is related to the serious renal insufficiency in HFRS. Both HFRS complicated with AP and ABP can cause pancreatic injury and inhibit insulin secretion, so the glycemia in most patients can be elevated, but there was no significant difference between the 2 groups.

Until now, there have been no effective drugs that can kill the Hantavirus.^[27]^ Because HFRS is generally a self-limiting disease, conservative treatment and close monitoring are recommended for these patients. Hemodialysis is an important measure for the treatment of HFRS in the oliguria period of acute renal dysfunction. According to the severity of the disease, hemodialysis can precisely control the volume load, stable blood flow, solute clearance rate, and filtration removal of the middle molecular cytokines and inflammatory mediators. Multiple organ functions and widely used hemodialysis can block the kidney damage process and significantly reduce mortality.^[28–30]^ In this study, 7 patients of HFRS complicated with AP were cured and discharged after conservative medical treatment and hemodialysis, which also demonstrated the above point of view. Recently, emergency endoscopic retrograde cholangiopancreatography, endoscopic Oddi sphincterotomy, endoscopic nasobiliary drainage, laparoscopic cholecystectomy, endoscopic US, and endoscopic stenting have been widely used in emergency biliary decompression and in drainage and removal of biliary obstruction, thus reducing biliary tract obstruction. Therefore, the occurrence of cholangitis and pancreatic necrosis is reduced, and the incidence of complications and mortality of ABP are greatly reduced. In our patient cohort, only 21 (20%) of 105 patients with ABP were discharged from the hospital after conservative treatment. Most of the remaining patients needed endoscopic or surgical intervention before their condition could be completely controlled. In our present study, there were certain proportions of complications (local or systemic) and mortality in the ABP group. Unfortunately, the statistical analysis was not carried out because the number of patients with HFRS complicated with AP was too small.

Although AP is seldom caused by the infection of Hantavirus, it should be considered if AP patients with acute renal insufficiency have a high fever before abdominal pain, especially during the prevailing season in the epidemic areas of HFRS. In addition, when HFRS patients present with abdominal pain, pancreatic enzyme levels should be detected, and imaging analyses should be conducted to assess the function and morphology of the pancreas to detect the complications of AP. Additionally, patients should also be asked whether they have other clinical symptoms, such as hemorrhaging (especially skin and mucous membrane), oliguria, and proteinuria, and pay more attention to the degree of leukocyte elevation, thrombocytopenia, and renal function indicators, such as creatinine, urea nitrogen, and other factors. This study has several limitations. First, these data were collected retrospectively. Second, the samples included in this study were not able to fully represent the statistical differences between the 2 populations. Therefore, this study requires a longer time, more central large sample data for statistical analysis, and more in-depth research. Third, this study only compares the clinical manifestations, laboratory tests, the treatment methods, and prognosis of the 2 diseases, but the pathogenesis and pathology of the 2 diseases were not studied. Thus, further studies, including more patients with HFRS complicated with AP, are warranted to understand the disease more thoroughly.

## 5 Conclusions

HFRS patients have many extrarenal complications, but they are less complicated with pancreatitis, resulting in pancreatitis being the rare cause of AP. ABP is the most common cause of AP, and there are many similarities in clinical manifestations and laboratory examinations, but there are also many differences, and the 2 treatment methods are very different. To reduce the misdiagnosis rate, clinicians should improve their understanding and differentiation in clinical work.

## Author contributions

**Conceptualization:** Jiang-Hua Yang.

**Data curation:** Jing Zhao, Ming-Yue Ni.

**Formal analysis:** Jin-Sun Yang.

**Investigation:** Wen-Jie Wang, Jing Zhao.

**Methodology:** Wen-Jie Wang.

**Supervision:** Jiang-Hua Yang.

**Validation:** Man-Man Liang.

**Writing – original draft:** Wen-Jie Wang.

**Writing – review & editing:** Wen-Jie Wang, Jiang-Hua Yang.
